# The prevalence of angina symptoms and association with cardiovascular risk factors, among rural, urban and rural to urban migrant populations in Peru

**DOI:** 10.1186/1471-2261-10-50

**Published:** 2010-10-08

**Authors:** M Justin S Zaman, Christian Loret de Mola, Robert H Gilman, Liam Smeeth, J Jaime Miranda

**Affiliations:** 1 Division of Medicine, University College London, London, UK; 2 CRONICAS Center of Excellence in Chronic Diseases, Universidad Peruana Cayetano Heredia, Lima, Peru; 3 Department of Medicine, School of Medicine, Universidad Peruana Cayetano Heredia, Lima, Peru; 4 Mental Health Working Group Peru, Universidad Peruana Cayetano Heredia, Lima, Peru; 5 Department of International Health, Johns Hopkins Bloomberg School of Public Health, Baltimore, MD, USA; 6 Department of Epidemiology and Population Health, London School of Hygiene and Tropical Medicine, London, UK

## Abstract

**Background:**

Rural-to-urban migration in low- and middle-income countries causes an increase in individual cardiovascular risk. Cost-effective interventions at early stages of the natural history of coronary disease such as angina may stem an epidemic of premature coronary deaths in these countries. However, there are few data on the prevalence of angina in developing countries, whilst the understanding the aetiology of angina is complicated by the difficulty in measuring it across differing populations.

**Methods:**

The PERU MIGRANT study was designed to investigate differences between rural-to-urban migrant and non-migrant groups in specific cardiovascular disease risk factors. Mass-migration seen in Peru from 1980s onwards was largely driven by politically motivated violence resulting in less 'healthy migrant' selection bias. The Rose angina questionnaire was used to record chest pain, which was classified definite, possible and non-exertional. Mental health was measured using the General Health Questionnaire (GHQ-12). Mantel-Haenszel odds ratios (adjusted for age, sex, cardiovascular disease risk factors and mental health) were used to assess the risk of chest pain in the migrant and urban groups compared to the rural group, and further to assess the relationship (age and sex-adjusted) between risk factors, mental health and chest pain.

**Results:**

Compared to the urban group, rural dwellers had a greatly increased likelihood of possible/definite angina (multi-adjusted OR 2.82 (1.68- 4.73)). Urban and migrant groups had higher levels of risk factors (e.g. smoking - 20.1% urban, 5.5% rural). No diabetes was seen in the rural dwellers who complained of possible/definite angina. Rural dwellers had a higher prevalence of mood disorder and the presence of a mood disorder was associated with possible/definite angina in all three groups, but not consistently with non-exertional chest pain.

**Conclusion:**

Rural groups had a higher prevalence of angina as measured by Rose questionnaire than migrants and urban dwellers, and a higher prevalence of mood disorder. The presence of a mood disorder was associated with angina. The Rose angina questionnaire may not be of relevance to rural populations in developing countries with a low pre-test probability of coronary disease and poor mental health.

## Background

Epidemiological work on coronary disease in the developed world has shown that much of the burden of cardiovascular diseases is attributable to environmental and lifestyle factors. The focus of such work has been on myocardial infarction rather than angina [1]. The burden of cardiovascular disease in low- and middle-income countries is increasingly of importance, [2-4] though there are few data on the global prevalence of angina in developing countries [5]. Progression from stable angina to myocardial infarction may be ameliorated by relatively low-cost interventions [6]. Hence, eliciting prevalence of angina and influences on its aetiology in low- and middle-income countries may allow targeting of cost-effective interventions at this early stage of the natural history of coronary disease, potentially stemming an epidemic of premature coronary deaths. Understanding the aetiology of an early cardiovascular phenotype such as angina is however complicated by the difficulty in measuring it, as traditional methods of measuring angina prevalence may be unreliable across different populations [7], and chest pain may signify psychological morbidity rather than coronary disease [8].

The environments of low- and middle-income countries are rapidly changing, becoming increasingly urbanised, [9,10] and the populations of such conurbations are typically larger than in developed countries [11]. Rural-to-urban migration in these rapidly-developing economies causes an uptake of unhealthy behaviours pertaining to increased future cardiovascular risk, [12,13] though few studies have examined prospectively the impact of environment change on onset of clinically-manifest cardiovascular disease. It is likely that the causal pathway (i.e., the cardiovascular risk factors involved) from urbanisation to cardiovascular disease is not vastly different in low- and middle-income countries as compared to the developed world [14].

We sought to examine prevalence of angina and influences on it within a rural-to-urban migration study in Peru. In particular, we wished to not only assess differences in prevalence in angina between rural, migrant and urban groups, but also assess how chest pain - both angina and non-anginal - associated with both classic cardiovascular risk factors and mental health/social capital. We used the previously validated Spanish version [15] of the 7-item Rose angina questionnaire, [16] the only standardised instrument for assessing typical angina that has been used extensively in developed countries, [17] though rarely in developing countries.

## Methods

### Study design

The methods have been previously reported [18]. Briefly, the PERU MIGRANT (PEru's Rural to Urban MIGRANTs) study, conducted in 2007, was designed to investigate the magnitude of differences between rural-to-urban migrant and non-migrant groups in specific cardiovascular disease risk factors. For this, three groups were selected: people who had always lived in a rural environment, those who had migrated from rural to urban areas and those who had always lived in an urban environment. Mass-migration seen in Peru from 1980s onwards was largely driven by politically-motivated violence rather than economic reasons, epidemiologically resulting in less 'healthy migrant' selection bias [18]. For the period 1988-1993, 50.7% of the total emigrants from the Ayacucho region moved to Lima, the capital of Peru, making Ayacucho the largest source of migrants to Lima. The village of San Jose de Secce, located in the Santillana district, Huanta province in Ayacucho was selected as the rural study site. Both urban and rural-urban migrant participants were selected from Las Pampas de San Juan de Miraflores, a peri-urban shantytown in the south of Lima.

### Participants

A single-stage random sampling method was used in all groups. For all study groups, individuals from both sexes aged 30 years-old and over, permanently living in their residence, were considered eligible. Pregnant women and those with a formal psychiatric health disorder that would impair survey completion were excluded. Participant selection was stratified by age-groups (30-39; 40-49; 50-59; >60 years old) and sex to ensure sufficient numbers of people in each stratum. Response rate at enrolment was 73.2%, and a detailed flowchart with response rates in each specific group has been previously reported [18].

### Data collection

A team of community health workers with previous fieldwork experience of household visitation enrolled participants. They were trained to conduct the specific questionnaires of this study and all assessments were made at the study coordinating centre. A demographic and socioeconomic survey collected age, sex and socioeconomic information. Systolic and diastolic blood pressures were measured using appropriately-sized cuffs for arm circumference in the sitting position using the right arm, supported at chest level. Three measurements at least five minutes apart using an oscillometric device (Omron M5-i, Omron, Japan) were made. The mean of the second and third systolic (SBP) and diastolic blood pressure (DBP) measurements were used for the analysis. All laboratory assessments were performed by trained personnel on venous blood samples taken in the morning after a minimum of eight hours fasting. The total cholesterol, triglycerides and HDL levels were measured in serum samples. In individuals with triglycerides below 400 mg/dL, LDL was calculated using the Friedewald equation in mg/dL (LDL = total cholesterol - HDL - (0.2 × triglycerides)) [19]. In individuals with triglycerides greater than 400 mg/dL, LDL was measured from the serum samples. Fasting glucose, fasting insulin and glycated haemoglobin were measured in plasma, serum and whole blood samples respectively. Waist circumference, measured in triplicate at the midpoint between the lower rib and the iliac crest, and hip circumference, measured in triplicate at the point yielding the maximum circumference over the buttocks, were also included. In addition, the 12-item General Health Questionnaire (GHQ-12) for mental health previously validated in Latin America [20,21] and a social capital instrument previously validated in Peru (SASCAT) [22] were also applied.

### Study variables

Hypertension was defined as SBP ≥ 140 mm Hg or DBP ≥ 90 mm Hg, or self report of physician diagnosis and currently receiving antihypertensive medication [23,24]. Diabetes was defined as fasting glucose ≥ 126 mg/dL (≥ 7 mmol/L) or self-report of physician diagnosis and currently receiving anti-diabetic medication [25]. Smoking status (never, former and current) was recorded using an adapted version of the WHO STEPS questionnaires [26]. A current smoker was defined as having smoked within the last six months and a lifetime total of more than 100 cigarettes.

Mental health was measured using the General Health Questionnaire (GHQ-12) [20]. The GHQ-12, though previously used in Latin American settings, has not been validated in Peru. However after consultation, a group of local experts concluded it was appropriate for this setting. The GHQ-12 score ranges from zero to twelve points, and was dichotomised; a score of five or more was considered a positive case for a mood related disorder (depression and anxiety), based on a previous study conducted in Santiago de Chile [21].

Social capital, defined as the social relationships, bonds and perceptions within societies or groups of people, [27] may also change after migration. Social capital was measured using the Short Social Capital Assessment Tool (SASCAT). The SASCAT questionnaire has been previously validated in Peru [28,29] and includes both a cognitive and a structural social capital component. In the cognitive component (trust, social harmony, sense of fairness) of the SASCAT, a score of three or more out of four points was considered as 'high' cognitive social capital [28,29]. The score in the structural component (group membership, involvement in citizenship activities and support from individuals in the community) was initially categorised in quarters, and subsequently made into a dichotomous variable with the lowest quarter as the reference.

### Exposures

The primary exposure was migration from a rural to an urban environment, defined by study group, i.e. rural, rural-to-urban migrant and urban groups.

### Outcomes

The Spanish-version Rose angina questionnaire was used to record chest pain in the standard way, and from this we classified chest pain into three mutually exclusive groups:

• "definite angina" if the pain was located over the sternum or in both the left chest and the left arm, was precipitated by exertion, caused the person to stop and went away in 10 minutes or less;

• 'possible angina", based on the 'possible' classification as originally proposed by Cook et al, [30] being chest pain brought on by exertion but not satisfying the additional criteria, such as location and relation to rest, necessary for a diagnosis of typical angina;

• 'non-exertional chest pain" defined as those complaining of chest pain that had no relation to exertion.

### Statistical methods

Prevalence estimates of non-exertional chest pain or possible/definite angina were calculated for each study group (rural, migrant, urban) and adjusted (age and sex, then with total cholesterol/HDL ratio, overweight or obese, smoker, diabetes, hypertensive and mental health). Mantel-Haenszel odds ratios were used to assess the risk of having pain in the migrant and urban groups compared to the rural group.

For general description of data, frequency analyses were calculated as number (percentages). Mantel-Haenszel odds ratios (OR) and 95% confidence intervals (CI) were used to assess the relation between risk factors and non-exertional chest pain or possible/definite angina after adjusting for age and sex.

### Ethics

Ethical approval for this protocol was obtained from ethics committees at Universidad Peruana Cayetano Heredia in Peru and London School of Hygiene and Tropical Medicine in the UK. The purpose of the study was explained to each of the study participants and informed consent was obtained, following international standards for ethical research in developing countries.

## Results

A total of 989 participants were included in the analysis. The mean age was 48.0 years (SD 12.0, range: 29-92), and 467 (47.2%) of the study group were men. There were 201 rural participants (born and living in Ayacucho), 199 urban participants (born and living in Lima) and 589 rural-to-urban migrants (moved from Ayacucho to Lima). Amongst migrants, the reported number of years of having living in an urban environment was 32 years (SD ± 10.5, IQR 25-39). The average age at first migration was 14.4 years (SD ± 8.5, IQR 10-17) whilst the average age on arrival into Lima was 15.5 years (SD ± 8.8, IQR 11-18). There was a good correlation between age at first migration and age on arrival into Lima (correlation coefficient = 0.92, p = <0.0001), thus indicating that migrants, who were born in Ayacucho, tended to move largely to Lima [18].

The prevalence of non-exertional chest pain was 2.5% for rural dwellers, 17.4% for migrants and 10.6% for the urban group (table 1) whereas possible/definite angina showed a different distribution, being most prevalent in the rural group (60.2%) followed by the urban and migrant groups (32.2% and 19.7%, respectively). Compared to the urban group, rural dwellers had an increased likelihood of possible/definite angina (multi-adjusted Mantel-Haenszel OR 2.82 95%CI (1.68-4.73) but a lower likelihood of non-exertional chest pain (multi-adjusted Mantel-Haenszel OR 0.22 (0.08-0.65)). Migrants had a lower likelihood of possible/definite angina (OR 0.47 (0.31-0.71)) and a trend towards a higher likelihood of non-exertional chest pain (OR 1.50 (0.86-2.62)) compared to the urban group.

**Table 1:** Prevalence and adjusted odds of non-exertional or exertional chest pain in migrants and urban dwellers compared to rural dwellers

	Group	Number (%)	Crude	Adjusted for age and sex	Adjusted for age and sex and CVD risk factors*	Adjusted for age and sex, CVD risk factors*, and mental health/social capital variables†
**Non-exertional chest pain**	rural	5 (2.5)	0.22 (0.08- 0.59)	0.21 (0.08- 0.58)	0.24 (0.08- 0.66)	0.22 (0.08-0.65)
	migrant	102 (17.4)	1.78 (1.08-2.93)	1.77 (1.07-2.92)	1.68 (1.01-2.82)	1.50 (0.86-2.62)
	urban	21 (10.6)	1	1	1	1
						
**Possible or definite angina**	rural	121 (60.2)	3.19 (2.12- 4.81)	3.31 (2.19- 5.03)	3.89 (2.43- 6.22)	2.82 (1.68- 4.73)
	migrant	116 (19.7)	0.52 (0.36-0.74)	0.52 (0.36- 0.74)	0.55 (0.38- 0.80)	0.47 (0.31-0.71)
	urban	64 (32.2)	1	1	1	1

Odds ratios and 95% confidence intervals; *CVD- cardiovascular risk factors -total chol/HDL ratio, overweight or obese, smoker, diabetes, hypertensive; †mental health (General Health Questionnaire) and social capital instrument (SASCAT).

Examining the prevalence of cardiovascular risk factors by study group, urban and migrant groups had higher levels of smoking, hypertension, diabetes, cholesterol and overweight compared to the rural group (Table 2). For example, 20.1% urban dwellers smoked compared to 5.5% of rural dwellers and 29.7% or urban dwellers were hypertensive compared to 11.9% of rural dwellers. When examining likelihood of pain by cardiovascular risk factors, participants with diabetes in the urban group were more likely to have possible/definite angina (OR 6.40, (1.52-26.8)), but there was no other similar relationship between possible/definite angina and other cardiovascular risk factors. No cases of diabetes were seen in the rural dwellers who complained of possible/definite angina. There was no relationship observed between non-exertional chest pain and any of the cardiovascular risk factors.

**Table 2:** Odds of non-exertional or exertional chest pain in those with risk factors for cardiovascular disease compared to no chest pain by migration status

Factor	Groups	Whole cohort	Non-exertional chest pain		Possible or definite angina	
**N**	rural	201	5 (2.5)		121 (60.2)	
	migrant	589	102 (17.4)		116 (19.7)	
	urban	199	21 (10.6)		64 (32.2)	
						
**Men**	rural	95 (47.3)	3 (60.0)	1.70 (0.28-10.4)	60 (49.6)	1.26 (0.71- 2.22)
	migrant	280 (47.5)	52 (50.1)	1.20 (0.78-1.84)	31 (26.7)	0.32 (0.21- 0.51)
	urban	92 (46.2)	12 (57.1)	1.61 (0.64- 4.02)	21 (31.8)	0.44 (0.24- 0.83)
						
**Current smoker**	rural	11 (5.5)	0	1.87 (0.30-11.5)	8 (6.6)	1.91 (0.47- 7.70)
	migrant	59 (10.0)	9 (8.8)	0.77 (0.36-1.66)	6 (5.2)	0.28 (0.65- 1.75)
	urban	40 (20.1)	7 (33.3)	1.85 (0.65-5.31)	11 (17.2)	1.48 (0.78- 2.55)
						
**Hypertensive**	rural	24 (11.9)	0	-	11 (9.1)	0.59 (0.23- 1.50)
	migrant	77 (13.1)	11 (10.8)	0.85 (0.42-1.73)	22 (19.0)	1.76 (0.96- 3.32)
	urban	59 (29.7)	5 (23.8)	0.94 (0.30-2.96)	21 (31.8)	1.12 (0.55- 2.30)
						
**Elevated cholesterol/HDL ratio**	rural	71 (35.3)	2 (40.0)	1.31 (0.21-8.16)	43 (35.5)	1.00 (0.55-1.83)
	migrant	328 (62.6)	64 (62.8)	1.06 (0.67-1.67)	71 (61.2)	0.94 (0.61-1.46)
	urban	123 (61.8)	12 (57.1)	0.81 (0.31-2.10)	42 (65.6)	1.47 (0.77-2.80)
						
**Diabetic**	rural	1 (0.5)	0	-	0	-
	migrant	13 (2.2)	1 (1.0)	0.41 (0.05-3.24)	5 (4.3)	2.22 (0.69- 7.16)
	urban	10 (5.0)	1 (4.8)	1.03 (0.12-8.80)	7 (10.9)	6.40 (1.52- 26.8)
						
**BMI overweight/obese**	rural	39 (19.4)	1 (20.0)	1.20 (0.12-11.90)	22 (18.2)	0.74 (0.36- 1.56)
	migrant	395 (67.3)	78 (77.2)	1.90 (1.14-3.15)	81 (69.8)	1.02 (0.65- 1.60)
	urban	141 (70.9)	11 (52.4)	0.43 (0.17-1.09)	50 (78.1)	1.59 (0.78- 3.23)

OR- Odds ratio; CI - confidence interval

Age and sex-adjusted; abnormal cholesterol/HDL ratio > = 4.0

Percentages in brackets

Compared to other groups, rural dwellers had a higher prevalence of mood disorder (49.0%) but a markedly higher degree of social capital in both of its cognitive (74.4%) and structural (92.0%) domains (Table 3). The presence of a mood disorder was associated with possible/definite angina in all three groups but not consistently with non-exertional chest pain. Amongst urban dwellers there was some suggestion that those with high cognitive social capital had lower odds of possible/definite angina, but there was no consistent pattern in the relationship between social capital and either non-exertional chest pain or possible/definite angina.

**Table 3:** Odds of non-exertional or exertional chest pain by presence of mood disorder and high social capital compared to no chest pain by migration status

Factor	Groups	Whole cohort	Non-exertional chest pain		Possible or definite angina	
**N**	rural	201	5 (2.5)		121 (60.2)	
	migrant	589	102 (17.4)		116 (19.7)	
	urban	199	21 (10.6)		64 (32.2)	
						
**Mood disorder**	rural	98 (49.0)	1(20.0)	0.20 (0.02-2.13)	66 (55.0)	2.39 (1.27-4.50)
	migrant	211 (38.1)	43 (46.2)	1.68 (1.05-2.71)	58(53.2)	1.64 (1.05-2.56)
	urban	62 (33.0)	3 (15.8)	0.35 (0.94-1.31)	29 (47.5)	2.34 (1.19-4.58)
						
**Structural Social capital (above lowest quarter)**	rural	185 (92.0)	5 (100)	1.02 (0.95-1.09)	114 (94.2)	1.68 (0.56-5.01)
	migrant	398 (68.2)	77 (75.5)	1.51 (0.92-2.47)	83 (72.2)	1.32 (0.83-2.10)
	urban	121 (61.7)	10 (47.62)	0.55 (0.22-1.40)	43 (69.4)	1.44 (0.75-2.79)

**Cognitive Social capital (high)**	rural	142 (74.4)	4 (80.0)	1.46 (0.16-13.51)	86 (75.4)	1.17 (0.60-2.28)
	migrant	266 (49.8)	44 (48.4)	0.92 (0.59-1.45)	55 (50.9)	1.13 (0.73-1.75)
	urban	77 (41.4)	7 (35.0)	0.66 (0.25-1.79)	18 (29.0)	0.48 (0.25-0.94)

OR- Odds ratio; CI - confidence interval

Age and sex-adjusted

Percentages in brackets

## Discussion

This study examined the prevalence of angina and influences on it in the context of rural-to-urban migration of a developing country. Our work has shown that, contrary to expectation, rural groups had a higher prevalence of definite or possible angina as measured by Rose questionnaire than urban dwellers. However, though diabetes was more prevalent in those urban dwellers with definite or possible angina, it was not so in those rural dwellers recorded as having definite or possible angina. A higher prevalence of mood disorder was observed in rural dwellers, mood disorder being associated with possible/definite angina in all three groups. Thus, a high level of mood disorder in the rural setting may explain much of the positive responses on the Rose angina questionnaire, rendering this measure of angina potentially invalid in such rural settings in developing countries.

These results can be potentially explained in a number of ways. Firstly, the Rose angina questionnaire may not be of relevance to rural populations in developing countries with a low pre-test probability of coronary disease. Indeed, 77% of the rural group did not have any risk factor (simple count of obesity, smoking, alcohol, hypertension, diabetes, high total cholesterol, data not shown) and in particular, none of the rural group with definite or possible angina had diabetes. Thus, the Rose angina questionnaire may be of more relevance to urban and migrant populations with higher global cardiovascular risk profiles. Secondly, the difficulty in measuring angina as a phenotype in differing ethnic populations was highlighted in a 2001 cross-sectional study in South Asian populations [7]. The Rose Angina Questionnaire was developed as a screening tool in Western countries and studies using it within developing world rural populations have shown differences in prevalence of angina by Rose questionnaire when compared alongside other prevalence measures of coronary disease [31]. In prospective studies however, those with angina ascertained by use of the Rose questionnaire have greater coronary mortality [32] and thus it remains to be seen whether the Rose questionnaire will have prognostic validity in developing countries. Thirdly, the use of a questionnaire to assess prevalence of angina may inadvertently pick up other conditions with similar symptoms. Anxiety disorders are particularly prevalent among primary care patients with chest pain [33]. In our study, the prevalence of having a mood disorder was greater in rural dwellers than in urban and migrant groups and was associated with definite/possible angina in all groups. Such a consistent association across groups was not evident for non-exertional chest pain, which as an entity did not relate to any study variable. Psycho-social factors such as depression and low-income have been shown to predict exertional Rose angina in a non-Caucasian population [34]. We also observed that a high level of cognitive social capital was associated with a lower prevalence of definite/possible angina within urban dwellers. This highlights the complexity of the relationships between cardiovascular risk factors, burden of coronary disease, population demographics and the social interactions of individuals within the populations of today's low- and middle-income countries.

### Strengths and limitations

Few other studies exist that seek to assess the prevalence of and aetiological drivers of angina, an important early stage of the natural history of coronary disease, in the developing world. The analyses presented in this study further challenge researchers on the appropriate measurement of clinical phenotypes in low- and middle-income country settings. This study thus adds to this growing field by highlighting the complexities and challenges for the assessment of angina in the developing world. We appreciate that a greater sample size would have made our findings more precise.

### Implications

Appropriate identification at incident symptomatic presentations of coronary disease such as angina may prevent or at least delay progression to more serious later manifestations of coronary disease such as myocardial infarction and coronary death if treatment is instigated at time of diagnosis. Whereas the measurement of a myocardial infarction is standardised through assays for biomarkers, angina is a clinical constellation of symptom descriptors, rendering quantitative measurement difficult. More discriminating measurement of angina symptoms, focusing more on the description of the chest pain, may complement existing angina assessment. Using a multi-centre chest pain clinic cohort, descriptors of chest pain using a simple symptom score were predictive of coronary events across sex and ethnicity [35]. A measure of the consistency of relation to exercise of chest pain may be a further discriminator in distinguishing those with and without coronary disease [30].

Our results also show the dangers of applying research results from the developed world to the health issues of low- and middle-income countries [36]. When facing the challenge of acting upon the epidemic of non-communicable diseases in developing countries, a measure which is of widespread use in one setting may have a different utility when applied to an alternative setting for which it was not designed for.

## Conclusions

Contrary to expectation, rural dwellers in Peru had a higher prevalence of definite or possible angina as measured by Rose questionnaire than migrants and urban dwellers. The Rose angina questionnaire may not be of relevance to rural populations in developing countries with a low pre-test probability of coronary disease.

## Competing interests

The authors declare that they have no competing interests.

## Authors' contributions

MJZ and JJM had the original idea. MJZ did all the statistical analysis, wrote the first draft and is the guarantor. CLdM and JJM were involved in further analysis. RHG and LS were involved in the discussion and interpretation. JJM designed and organised the cohort and was involved in the discussion and interpretation. All authors participated in the discussion and interpretation of the final results and contributed to the final paper.

## Pre-publication history

The pre-publication history for this paper can be accessed here:

http://www.biomedcentral.com/1471-2261/10/50/prepub
